# Analysis of the miRNA targetome in EBV-infected B cells

**DOI:** 10.1186/1750-9378-7-S1-O2

**Published:** 2012-04-19

**Authors:** Rebecca L Skalsky, David L Corcoran, Eva Gottwein, Christopher L Frank, Markus Hafner, Jeffrey D Nusbaum, Regina Feederle, Henri-Jacques Delecluse, Micah Luftig, Thomas Tuschl, Uwe Ohler, Bryan R Cullen

**Affiliations:** 1 Department of Molecular Genetics and Microbiology, Duke University, Durham, NC, USA; 2 Duke Institute for Genome Sciences and Policy, Duke University, Durham, NC, USA; 3 Department of Microbiology-Immunology, Northwestern University, Feinberg School of Medicine, Chicago, IL, USA; 4 Laboratory of RNA Molecular Biology, The Rockefeller University, New York, NY, USA; 5 German Cancer Research Center, Department of Virus-associated Tumours, Im Neuenheimer Feld 242, Heidelberg, Germany; 6 Department of Biostatistics and Bioinformatics, Duke University, Durham, NC, USA

## 

microRNAs (miRNAs) are ~22 nt, non-coding regulatory RNAs expressed by all metazoans and several viruses. During latent infection, Epstein-Barr virus (EBV) expresses 25 pre-miRNAs and influences the expression of cellular miRNAs, such as miR-155 and miR-21, all of which potentially have roles in viral oncogenesis. To date, only a limited number of EBV miRNA targets have been identified; thus, the role of viral miRNAs in viral pathogenesis and/or oncogenesis is not well defined. Using photoactivatable ribonucleoside-enhanced crosslinking and immunoprecipitation (PAR-CLIP) [1] combined with high-throughput sequencing and computational analysis [2], we interrogated the miRNA targetome in EBV-infected B cells. We identified miRNA binding sites in over 5,700 cellular 3’ untranslated regions (UTRs), 25% of which contained sites for EBV miRNAs. miRNA binding sites were also identified at a lower frequency in coding regions. Our results reveal that EBV miRNAs predominantly target host cellular transcripts, thereby reshaping the host environment. Furthermore, viral miRNA targets are involved in multiple biological processes that are directly relevant to EBV infection, including modulation of immune responses, cell proliferation, and cell survival. Finally, we identified a number of viral transcripts that contained conserved binding sites for cellular miRNAs, including members of the myc-regulated miR-17/92 cluster. This comprehensive survey of the miRNA targetome in EBV-infected B cells is a positive step towards identifying novel therapeutic targets for EBV-associated malignancies.
